# Report on Delirium Tremens

**Published:** 1856-10

**Authors:** John Barttett

**Affiliations:** Keokuk, Iowa


### A REPORT ON DELIRIUM TREMENS, READ BEFORE THE LOUISVILLE MEDICAL CLUB, FEB., 1856.

BY JOHN BARTTETT, M. D. KEOKUK, IOWA.

Investigations as to the functions of the nervous system, made of late years, have added much interest to the study of disordered brain action.

The recognition of a chain of ganglia at the base of the brain in man, similar in function to the ganglia of invertibrate animals,as the seat of consciousness of the individuals—as the centre of afferent and efferent forces, sensory and motor, sensational and intellectual ; and the establishment of the exclusively intellectual function of the gray matter of the cerebral hemisphere, have led to the classification of many facts which once defied generalization. The developments aid us somewhat in the study of Delirium Tremens.

Alchoholic stimulants, when taken internally, enter the circulation, reach the brain substance, and in a moderate dose stimulate the whole organism. An increase of the dose oppresses certain portions of the brain, while it continues to stimulate others—thus the cerebellum and sensory ganglia, the muscular movements and senses, appear to be confused before the intellect has lost its quickness. Upon further impression the sensory ganglia seem oppressed, and although certain functions of the cerebrum may be still active—as the imagination it will be noticed, that perception is becoming less active, that the will is losing control over the train of thought, and that the reason vanishes. In the state of complete intoxication, the susceptibility of the ganglia of external relation is quite lost: though the cerebral ganglia still send impressions to the sensorium, so that with powerlessness of the muscular system there may be a dream-like dwelling of the mind on past events ultimately the cerebral ganglia themselves may become inactive, and the consciousness of the intoxicated man is impressed neither objectively nor subjectively, he is completely anæsthetizised—perfectly comatose. The Alchohol next affects the spinal cord and medulla oblongata, producing convulions and death.

Alcohol is not confined in its action to the nervous system; it affects doubtless every organ of the body. The stomach and intestines, the liver, kidney, and skin are all influenced by it.

Now in a system, habituated, in a certain degree, to the use of alchohol, there occurs, upon the withdrawal of the stimulus, a derangement of he same nerve centres, and organs which are affected in alcoholic intoxication, and this derangement constitutes Delirium Tremens. Disorder of the stomach and intestines, of the liver, kidney and skin, are well marked in most cases. But alcohol has a peculiar affinity for the nerve substance, as is shown by its presence in the brains of animals, poisoned by it, by its presence in the serum of the ventricles, and especially by the fact of its existing more abundantly in the nerve substance, than in the blood itself, and it is this affinity which causes the brain to present the prominent symptoms of the disease. Derangement of the sensory ganglia is apparent from the muscular tremors and disordered sensibility—from the spectral illusions, imaginary sounds, and impaired taste. Derangement of the cerebrum furnishes the characteristic symptoms of the disease.

*Nature.*—Most every writer on this disease has speculated as to its nature; a few of the more plausible theories may be profitably presented.

Dr. Tweedie states that in the sthenic form of the disease, the congestion is arterial, while in the asthenic forms, it is venous, and, that both kinds of congestions, by producing partial pressure and augmented action, may give rise to cerebral symptoms of the same character.

Drs. Clutterbuck and Bright regard the sthenic form as an inflammatory action in the archnoid and pia mater.

Drs. Copland, Stokes, and Wood believe that exhaustion and depression of both the nervous and sensorial powers are necessary for its supervention.

Drs. Ware and Geshard regard it as a specific disease, like measles, scarlatina, etc., having a definite course and a natural termination.

Dr. Joseph Klapp is the advocate of the gastric pathology. He asserts that dissection almost uniformly discloses traces of previous inflammation of the stomach, and that almost invariably a foul tongue, nausea and vomiting are present. That the operation of an emetic almost always causes the rejection of a viscid, light brown, or black colored fluid, of the consistence of tar, and that he has observed relapses following immediately upon indiscretion in diet, and re-establishment of convalescence succeed to the rejection of the offending substance.

Dr. Blake regards the disease as a paroxysm. He compares the first stage, that of exhaustion, to the cold stage of ague—the second, or that of arterial excitement, to the hot fit—and the sleeping, or third stage of this disease, to the sweating stage of intermitting fever. This paroxysmal effort, he thinks, may be instituted by the nervous system to compensate for the loss of stimulus consequent on the sudden cessation of intemperate habits. "We thus," he says, "are enabled to understand how the arterial system at first sympathises in the nervous depression, and afterwards in its reaction, and finally, how it participates in the calm which is restored during the supervention of the sleeping, or third stage."

Dr. Stokes has witnessed a good many dissections, and among the most common appearances were unequivocal marks of inflammation in the brain and stomach. All sthenic cases, arising from bouts in those not much accustomed to intoxication, he regards as instances of gastritis.

It has been the theory of almost every writer on Delirium Tremens, that it was the deprivation of stimulus that brought on the disease—that tippling was its predisposing, and deprivation its exciting cause. This theory began to be questioned a good many years ago, and in order to sustain it, its supporters have been obliged to admit varieties of the disease. As far as my information extends, Dr. John Ware, of Boston, was the first to affirm that in the larger proportion of cases, abstinence has nothing to do with inducing the disease. And it is remarkable that he offers no facts or arguments to substantiate this extraordinary position. These, however, have not been wanting to Dr. Peddie, of Edinburg. He tells us that Dr. Page, Surgeon to the county jail of Carlisle, states, that although the annual number of commitments to the prison during the last fifteen years has been about six hundred, and although three-fourths of these arrests were considered to have been in one way or another the consequence of drunkenness, he has never yet seen any ill results from the sudden abstraction of stimulants from habitual drunkards, who had been drinking to excess up to the time of being placed on prison fare.

Dr. Scott, of the jail of Dumfries, states that during the last fifteen years, the number of prisoners has amounted to five thousand five hundred and thirty-nine; that of this number, he supposes that about two-thirds were admitted for crimes resulting from intemperate habits—that he believes a very large number to have been habitual drunkards, and that although all of these, of course, were deprived of their usual libations, and at once put on prison allowance, only five cases of Delirium Tremens are found on the register of disease, and that all of these patients but one were admitted with the disease on them.

Dr. Gibson, of the prison of Glasgow, shows that while four thousand one hundred and twenty-two were imprisoned, the number of assaults committed under the influence of liquor amounted to one thousand five hundred and nineteen, and of this number only three case of Delirium Tremens occurred.

Dr. Simson, the medical officer of the prison of Edinburg, states that the whole number of prisoners committed last year was five thousand eight hundred and sixty-four, and only four cases of Delirium Tremens occurred during the last eighteen months. The average number of cases, during former years, was two or three per annum. Dr. S. considers that at least one-half of the prisoners were dissipated characters, and that at the very lowest computation, five hundred must have been regular systematic drunkards, from whom all drinks were suddenly abstracted. In the four cases reported, the patients had symptoms when admitted.

To Dr. Peddie it is apparent that habitual excess in the use of stimuli is alike the exciting and predisposing cause of Delirium Tremens, and that, if a suspension or diminution of habitual supplies be, at any time, attended by symptoms of the disease, these are not to be regarded as resulting from the change in the quantity consumed, but as occurring in spite of such change, and because the peculiar constitutional effect has already been induced, and the premonitory stage of the affection already begun.

The facts collected by Dr. Peddie, though on some accounts remarkable, do not sustain his position, and the statement of Dr. Joshua B. Flint, formerly Physician to the House of Correction, of Suffolk county, Mass., seems fatal to it. Dr. Flint states that during the six or seven years of his attendance at that institution, at least one thousand cases of Mania-a-Potu occurred, and that in nearly all of these instances, the disease was developed after confinement, and the abstraction of stimulus.

The present discrepancy as to the symptoms, but especially as to the circumstances under which Delirium Tremens originates, positively prevent the construction of any theory which will cover the conditions presented by each writer. Every observer of the disease must then form his own idea of its nature, rejecting such statements as untrue, which are directly antagonistic to the results of his own repeated observation.

Dr. R. B. Todd, who has written so much and so well on Delirium and Coma, regards the delirium of Delirium Tremens as toxic, as depending on the introduction of alcohol into the blood. He considers it essentially humoral in its origin. The poison he regards as partly alcohol, and partly a material derived from a depraved and destructive assimilation of the brain itself. The existence of this poison, distinct from alcohol but generated in consequence of its habitual ingestion, will explain the production of delirium, in the absence of the accustomed stimulus.

I regard the delirium as chiefly toxic, and although the formation of a new poison, as assumed by Dr. Todd, is not irrational, it seems unnecessary. To me the toxic agent seems to be the blood itself. If alcohol is taken by a temperate man, his blood is poisoned; it is so altered as to produce delirium. Now in habitual drinkers the pabulum of the brain is not normal blood, but blood and alcohol. Change the character of their blood and you produce delirium. Deprive an habitual drunkard of his stimulus, and as the alcohol within him is eliminated, the brain is starved, till in twenty-four hours, or a week, the quality of its nourishment so differs from its wanted pabulum, that disordered action ensues.

This condition, which constitutes the disease in its incipiency, is followed by a constitutional disturbance, which forms part of the phenomena of the disease in the subsequent stages. In starvation, delirium and convulsions sometimes occur, and such is the constitutional derangement, that the customary food, if not cautiously given, produces delirium. Those diseases in which the tissues are deprived of their supply of healthy blood, as scurvy and anæmia, furnish examples of cerebral derangement. But the pathology is not altogether humoral. The drunkard's ability to take an inordinate amount of liquor is dependent upon the accommodation of his system to its influence, and that accommodation continues to exist, to a greater or less extent, though the old habit be long given up. Delirium Tremens can only occur in a person having the Ebriositatic Diathesis. Now this diathesis, like the tolerance of liquor, is never entirely lost, and cases occur, when, after a long abstinence, Delirium Tremens is induced by a debauch, altogether insufficient to occasion it, without the peculiar condition resulting from his former habits. It is plain, therefore, that there is a change in the brain in these cases, and that this change is more or less permanent. Nor is this alteration in tissues, by which alcohol becomes the necessary pabulum, confined to the brain. It affects every organ; and, in fact, these changes are more observable in some organs than in the brain. Dr. Oysten, of Aberdeen, has shown that in drunkards, almost all the organs in which alteration can be detected, present some lesion. There is, I doubt not, in Delirium Tremens, a derangement of the liver, stomach, kidneys and skin, corresponding, in some degree, to that of the brain.

The symptoms of the disease form the history of this derangement. We see that it is quite regular in frequency and march, and that in the great majority of cases, it tends to a favorable termination. The views of the nature of the disease, as expressed by the authors I have quoted, are pretty generally founded upon their views of the pathology. In the cases which I have examined, congestion of the veins of the dura mater, slight opacity of some portions of the arachnoid, with occasional arachnoid, and intraventricular effusion were the only lesions found. These, and some others, have been noticed by all examiners. Dr. Hoegh

Guldberg describes an effusion of lymph, under the arachnoid, as found frequently in the bodies of robust young men. Dr. Stokes frequently finds gastritis and meningitis in sthenic cases. I regard these lesions as complications—and most of those described, as having little connection with the disease. Dr. Oysten's examinations of the bodies of one hundred and seventeen whisky drinkers show that all those pathological appearances which have hitherto been regarded as at least coincident with Delirium Tremens, are found in the bodies of drunkards dying suddenly, while apparently in ordinary health and activity.

*Treatment.*—According to Armstrong, opium is the main remedy for Delirium Tremens, especially when there is a soft, compressible pulse. He gave three or four grains at once, and afterwards, two grains every four, five or six hours. He advises this plan to be continued forty-eight hours. It will succeed in that time, if at all; and if it fail, it is hazardous to push it further.

Dr. Hoegh Guldberg, of Copenhagen, bleeds from the arm occasionally, in young patients, having, what he regards, symptoms of arachnitis. But he thinks ℥xii sufficient, and in most cases local blood-letting had better be substituted. In young and strong persons, he uses frequently, cold applications and affusions. They dissipate hallucinations better than any other remedy, in the first stage, and to this stage he confines their use. He repeats the affusion every two hours, regardless of the state of the skin. In the asthenic form, Dr. Guldberg, considers opium the main remedy, but he never gave it to a greater extent than forty-eight grains in any case.

Dr. Coates says that sleep must be produced, *coute qui coute.* He thinks that every case of simple Delirium Tremens may be cured by opium, and he states that he never saw, heard, or read of an instance, in which opium was productive of harm.

Dr. Carter regards purgatives as necessary, and of them, he prefers croton oil. He thinks that a liberal exhibition of the accustumed stimuli in the incipiency, will be likely to induce the second stage. But opium is the one great and powerful remedy.

Dr. Eberle regards opium as the remedium magnum. But, it has appeared to him, that if used before the intestines are freed of their vitiated contents, its free employment has a tendency, in some instances, to cause dangerous determination to the head, and to bring on coma instead of sleep.

Dr. Copland directs, in the species with increased vascular excitement, moderate depletion with cups or leeches, and cold affusion to the head, whenever the temperature rises above the natural standard. Upon the subsidence of the vascular excitement, the consequent depression should be met by moderate doses of opium. During the first period of the second stage, an effort should be made to cut short the disease by opium, or opium, camphor and carbonate of ammonia, and by paying attention to the liver and bowels. He regards opium as necessary in the cure of Delirium Tremens, as bark in the cure of Ague.

Dr. Tweedie directs, in the form with strong pulse, hot head, and flushed face, that the congestion be abated by general and local bleeding, or, when these means are contra-indicated, by cold affusion to the head. In the asthenic form, he says the symptoms generally yield to opium, camphor, ammonia, and sulphuric ether.

Dr. Blake uses the stimulo-narcotic treatment in the incipiency. When the disease is established, he adopts the full opium treatment. As a purgative, he prefers croton oil. In his opinion, it would be as vain to attempt to arrest this disease, when fully formed, as to stay the progress of Typhus Fever.

Dr. Watson regards opium as the great remedy, but cautions against its indiscriminate use. In some cases, it must be combined with depletion, and in others, with stimulants.

Dr. Wood says, there is no easier or more certain method of removing the delirious symptoms, and restoring the patient to his previous state of health, than to resort to the accustomed stimulus. But moral considerations deter him from this course, and he uses opium, regarding it as an excellent substitute.

Dr. R. B. Todd insists on the importance of producing sleep, and he prefers to use crude opium. To remove irritability of the stomach, he knows no medicine that succeeds better than quinine and opium. One grain of the one and half a grain of the other, every four, six or eight hours. When the pupil has very much contracted, he would substitute chloroform for the opium. And in this contra-indication, Dr. Holland suggests the use of the opposite class of narcotics.

Dr. W. S. Macgregor, of India, regards the condition of the pupil as of the utmost importance in the management of the treatment of Delirium Tremens.

Another mode of treatment of Delirium Tremens, which has excited much attention, is the emetic plan. This was first used by Stoll, in 1728. Goden and Spence gave tartar emetic, the latter in large and frequent doses, and with happy results. But Dr. Law, of Dublin and Dr. Klapp, of Philadelphia, first brought this practice into general notice. They gave two grains of tartar every fifteen minutes, till vomiting occurred, when an immediate amendment resulted. Dr. Klapp reported eighty-one cases and all recovered but one patient, who died of epilepsy. This practice, although on the whole, promising much better than the opium plan, has not been generally adopted. In some cases, it seems to be contra-indicated by the feebleness of the patient, and it is not without danger. Dr. Eberle reports two deaths from the prostrating effects of tartar emetic in subjects in which such a result was not to be apprehended. Similar cases have since occurred. The emetic plan has been much practiced at the European General Hospital at Bombay, and it has proved more satisfactory than the opiate treatment. Dr. Peddie, of Edinburg, relinquishing opium, has adopted the emetic plan. He refers to eighty cases, some of them very severe, all of which were speedily cured by that method.

Dr. Graves, of Dublin, has long considered antimony a soporific. He frequently combines it with opium in the delirium and insomnia attending the latter stages of typhus, and he uses the same combination in Delirium Tremens, with remarkable success.

This compromise of the emetic and opium plans of treatment, is fast gaining ground among us. Dr. R. B. Todd suspects that in giving tartar and opium, we may simply be giving opium. This, however, is a groundless suspicion. The opium moderates the depressing influence of the tartar, without suppressing its eliminating effects, and the tartar renders the system much more susceptible to the narcotic.

Bleeding was at one time, under an erroneous view of the pathology of the disease, highly recommended as a curative agent. In a few of the cases it succeeded pretty well, but it has justly fallen into disuse. Dr. Sutton stated that when blood-letting had been principally relied on, he had observed a fatal termination in almost every case. And all the patients that Dr. Armstrong saw copiously bled, died. The testimony of Dr. Stewart is to the same effect. Dr. Clutterbuck, writing in 1846, says the antiphlogistic course should be pursued in Delirium Tremens. He recommends blood-letting to the very moderate extent of two or three ounces. He cites cases, showing the success of his plan, when opium had failed.

Another mode of treatment consists in the continued administration of the accustomed stimulus. Dr. Michael Reryan seems the first to have called attention to this method in 1828.

Dr. Gerhard furnishes us a summary of the results of treatment at the Philadelphia Hospital, in a series of years. The plan of treatment at first pursued, was confinement, and more or less active use of opium. Other adjuvant means were not, however, dispensed with, as cups, cold applications to the head, purgatives, &c. This mode of treatment was gradually abandoned, and the stimulating plan adopted. In the year ending in May, '35, the mortality was one in eight, the next year one in nine; for the third year, one in six—an increase in the mortality, depending upon the prevalence of an epidemic of typhus, which attacked many of the debauched patients. In the fourth year, the mortality was one in eleven, and this amelioration coincided exactly with the diminution of the doses of opium. In the fifth year, the deaths were one in twenty-six; in the sixth, one in one hundred and sixty-one; and up to Aug., '41, the mortality, including complicated and moriband cases, was about one per cent. Of the accustomed stimulus, Dr. Gerhard gives as a dose, the usual allowance of the patient when in health. It is necessary to state that Dr. G. attributes this diminution in the mortality, in a good degree, to the change in the regimen, which was made coincidently with that in the treatment. Instead of confinement, the patient was allowed as much liberty as possible.

Dr. Kuhn of Philadelphia, treated his patients by confining them in a dark cell, and leaving the disease "spontaneously to work itself off." And this he found to answer better than any other plan of treatment. The most important fact yet discovered relative to the management of delirium tremens, is here disclosed, *that the disease tends to recovery.* The expectant plan has been fairly tried since the time of Dr. Kuhn, and with nothing, as far as I am informed, but the most satisfactory results. Dr. Hoegh Guldberg treated twenty-two cases on this plan, and twenty-two recovered. Dr. John Ware treated twenty-nine cases in the simplest way, and of these only one died, and in that one arachnitis was revealed upon dissection. Dr. Joshua B. Flint, of this city, was, about the year 1835, Physician to the House of Correction, for Suffolk county, Mass. Many drunkards were brought in here, and many cases of delirium resulted from the abstinence enforced. These cases were locked in a cell with padded walls. They were allowed plenty of worm-wood tea and good soup—and nothing else. In one year there occurred two hundred and sixty cases, and of these only two died. Both of these patients accidently lost blood from injuries received. Dr. Flint was physician to this Institution for six or seven years, and his experience, during the whole term, was not unlike that given above.

In addition to these established modes of treatment, some physicians give exclusively purgatives, chiefly calomel and croton oil. Many other articles of the Materia Medica are recommended, digitalis, prussic acid, Indian hemp, belladonna, ammonia, &c. The testimony in favor of these is of little value, seeing that it is not shown that the results from them are more favorable than those furnished by the expectant treatment.

I shall not occupy time with the detail of my own course of treatment. In the incipient stage of an attack, brought on from abstinence, without a preceding debauch, it may almost always be arrested by a recurrence to the accustomed stimulus. And we are more certain to secure this result, by adding moderate doses of opium to the drink. Even when the disease is fully formed, when certain that it is but just established, I attempt to jugulate it by the same means. But this effort is not continued longer than twenty-four hours, unless it is promising very soon to succeed.

During the elimination of the excess of alcohol, and the correction of the disordered functions following immediately upon a debauch, some benefit may be derived from leeches or cold to the head, and from the application of wet or dry cups, or of a blister to the stomach, and from the use of ice and carbonic acid. But it is of importance, if the patient be of the ebriositatic diathesis, that there should be a continuance of the customary drink. I am aware that Dr. Stokes is Broussaist enough to regard the delirium following these disturbances, as the result of gastritis, and that he has long treated delirium tremens so arising, with leeches to the epigastrum, ice, &c. But his success in this way has been no greater than has followed the practice of those who do nothing at all. Leeches to the stomach and ice relieve, it may be delirium and tremor, almost instantly. But such relief has sometimes followed the abstraction of a like quantity of blood from the head. Dr. Stokes dwells upon the sympathy between the stomach and the brain. Does he forget that this sympathy is reciprocal.

Dr. Gerhard regards this condition of the stomach as inflammatory, but he does not suspend the stimulus on that account. He finds, as Dr. Stokes did, in reference to chicken soup, in certain stages of gastritis, that there is no inconsistency, and there is much benefit, in the practice. When the stomach continues irritable, I should attempt to ward off the disease by opium. If delirium continue in spite of these means *it must run its course.* Throughout the disease, I pay attention to the stomach and bowels, an occasional emetic or purgative is administered; nourishing broths are regularly given except when there is too much febrile excitement or irritability of the stomach—and as little restraint as is consistent with safety is used. If the patient is quite unmanageable, tartar emetic will subdue, without injuring him.

But, if the disease progress, and no rest is obtained in five days or a week, and if symptoms of the third stage supervene, I have not refrained from the administration of opium and alcoholic stimulants, nor from the application of blisters to the head. It is impossible to say whether this interfeience is judicious.

I believe with Ware, Blake and others, that delirium tremens is a self limited disease, but I differ from them in believing that its progress may be arrested. I am sure I have observed such an effect from the use of opium frequently, and it is a question whether, when we are apprehensive that the disease is about to terminate ill, and when the treatment, or such part of it as we have directed, has been thus far expectant, an effort should be made to induce sleep by opium; I think I have seen such efforts in such circumstances, successful. There is another article which promises very well in this condition of things. I refer to Chloroform. I have read the report of six cases treated by anæsthetics, three by ether and three by chloroform. In these cases these articles were used on the first, third and fourth days of the disease, and after the free use of opium—the general result was an immediate sleep of some hours, terminating in restoration of the mental faculties. This fortunate result occurred in every case, but in some instances the inhalation was several times repeated.

Dr. Corrigan, of Dublin, considers its use by inhalation extremely dangerous, and mentions a case almost lost by asphyxia. Five cases terminating fatally have come to his knowledge; in one, death was instantaneous, in the second the patient sank in less than an hour with blue lips and cold surface. Death followed immediately upon its administration by Prof. C. L. Lyle, at the Louisville Marine Hospital last year; the patient was an old drunkard, and in the last stage of the disease.

I have noted ten cases of its internal administration in delirium tremens. It was used in most of these cases about the time of the coming on ot the critical sleep, though there was in no case an evidence that it was about to supervene. In one instance it seemed to jugulate the disease, and in all, its effects were so decided, that I have no doubt of its agency in producing or anticipating the sleep. It has been given in both sthenic and asthenic form of the disease.

In the coma of the last stage, perhaps blisters have been too much neglected. Dr. R. B. Todd says, the tendency to coma depends on the non-elimination of urea and other elements of the urine, and he directs blisters to the head and neck as a means of eliminating the poison.

The Prognosis of the disease depends almost entirely upon the treatment. Without treatment, the termination in the great majority of cases, is favorable. With some form of management, it proves more fatal than inflammation of the brain. Of 3891 cases, collected from different sources, and treated by different methods, 452 died—showing a mortality of 1 in 8.6. During the six months, ending October '51, I observed at the Louisville Marine Hospital, the treatment of a number of cases by opium, and the straight jacket. For two months, opium was used in heroic doses. In several of the worst cases, ʒss of laudanum was given every half an hour—nor was the presence of coma or convulsions regarded as a contra-indication. During the remaining four months the opium was the magnum remedium, but it was used with some moderation, and some attention was paid to the state of system; 32 cases were recorded as delirium tremens, of these, 16 were mild, yielding to opium and stimulus, and being discharged in two or three days. The remaining 16 were severe cases, and of these 14 died.

At the same Hospital for 5 years ending March 10, 1855, 271 cases were admitted, of these 72 died—a mortality of 1 in 3.8—Opium has been the remedy mostly relied upon, and the straight jacket has been freely used.

Diagnosis.—Dr. H. Bence Jones, has afforded us an excellent means of distinguishing this disease from that which it most simulates, and from which it is most important to distinguish it, Inflammation of the Brain, and Epleptic Delirium. He says: "In chorea and delirium tremens, and in these alone, do I find the quantity of sulphates in the urine exceedingly increased, while at the same time the quantity of the phospates is not increased at all."

Remarks by Members of the Club.—Dr. S. D. Gross had seen a good many cases. He depended mainly upon opium, generally combined with tartar, in the sthenic forms. In the asthenic cases, he combines it with carb. ammonia, giving 10 or 15 grs. at a dose. In the later stages, camphor mixture and opium, is a favorite prescription with him. He depricates general blood letting. When there is much excitement it is quieted by antimony and cold effusions. In first attacks very little need be done. He had seen more deaths from the *treatment* of delirum tremens than from the *disease*. We are apt to do too much. He had used chloroform by inhalation, with advantage in several cases.

Dr. John Hardin, when in the country, treated successfully three cases, ensuing immediately upon a debauch. He bled from the arm, and afterwards poured a dozen gallons of cold water upon the head. The effusion calmed the patients immediately; and it was repeated several times in each case. He has seen in city practice no cases demanding the lancet. He relies upon morphine and tartar. In some feeble persons he has given morphine alone, but, even in these, in some instances, he had resorted to tartar with the happiest results, after the morphine had failed to produce sleep. He had never known sleep fail to follow relaxation by tartar.

Dr. B. I. Raphael in the premonitory symptoms, gives an emetic or cathartic; after vomiting, he orders ℨi tinc. of lupuline to be put in a bottle of porter and, directs that a draft of this be given whenever the patient desires to drink. When the case is more advanced, he uses opium and tartar. In his practice he does not think the mortality is higher than one in twenty.

Dr. T. G. Richardson had seen more cases get well under a moderate use of whiskey and laudanum than under any other treatment.

Dr. J. Knight concurs with Dr. Gross. He gives tartar in small doses with laudanum. In asthenic cases he gives ammonia.

Dr. Wible, said the secretions should first be established. He thinks favorably of tartar, and he gives opium with it. He has treated fifteen or twenty cases, of these three died.

Dr. B. R. Palmer, saw immediately fatal results, apparently from treatment in two cases. In one case, the man was rushing about the room furiously delirious: he was bled, and died very soon afterwards. He saw chloroform given to a stout man laboring under delirium tremens. This man died whilst the chloroform was being administered, and when but little had been inhaled.

Dr. W. B. Caldwell had treated several cases with opium and tartar; all recovered.

Dr. N. B. Marshall's experience was confined to milder cases. His success had been good. He gave calomel and jalap, and afterwards quinine and opium. This combination produces a sedative effect, and acts more favorably than opium alone.

Dr. Goldsmith had great confidence in the tartar emetic plan. He treated an opium-eater for mania-a-potu, six times; giving 5 or 10 grains of tartar at once. This dose acted kindly, and in a day or two the patient would be out. In one case, occurring in a very robust man, he gave 31 of tartar emetic in 12 hours. It produced active vomiting and purging, followed by sleep and convalescence. In a few days he was well. He never saw ill results from tartar emetic.

Dr. Lewis Rogers had seen many cases. He regards it as induced by the withdrawal of stimulus. He considers it a state of exhaustion, and he gives alcohol. He has made trial of the other remedies recommended, and they had not proven satisfactory. Occasionally, he combines some narcotic with the stimulus, and some cases require opium. To one patient he gave 28 grs. of tartar: fatal prostration supervened. There was no emesis but frequent choleraic discharges. Cold water to the head is serviceable. Dr. Rogers is satisfied that active treatment is hurtful. He has seen many patients killed by opium.

Some remarks were made by gentlemen, relative to the occurrence of the disease during a debauch, and not connected with abstinence or privation; Drs. Harden, Palmer and D. W. Yandell, had seen it occur under such circumstances. Dr. Hardin said that it was possible that the abuse of good whiskey was not followed by delirium tremens. He had known many sots in the country, who were for many years intemperate, without the occurrence of mania.

Dr. Yandell had, in one case, seen delirium tremens produced by drinking hard cider.

Drs. Goldsmith and Caldwell, mentioned each a case, resulting from the deprivation of tobacco. Excess in the use of coffee was mentioned as a cause.

Dr. D. W. Yandell mentioned a case in which he poured eight or ten buckets of cold water upon the head. In ten minutes the patient died. In another instance, he administered chloroform, and the patient expired in less than half an hour.

